# Identifying delay in glymphatic clearance of labeled protons post-acute head trauma utilizing 3D ASL MRI (arterial spin labeling): a pilot study

**DOI:** 10.1038/s41598-024-56236-6

**Published:** 2024-03-14

**Authors:** Charles R. Joseph, Jubin Kang Lim, Bryce N. Grohol, Marija Zivcevska, Joshua Lencke, Ethan Dean Rich, Connor James Arrasmith, Ian Shepherd Dorman, Bradley Waman Clark, Kim Love, Ben Ferry, Mark E. Rolfs

**Affiliations:** 1 Liberty University College of Osteopathic Medicine, Lynchburg, USA; 2 K. R. Love Quantitative Consulting and Collaboration, Athens, USA

**Keywords:** Neuroscience, Biomarkers, Medical research, Neurology

## Abstract

This study correlated mild traumatic brain injury (mTBI) cognitive changes with ASL-MRI glymphatic clearance rates (GCRs) and recovery with GCR improvement. mTBI disrupts the blood brain barrier (BBB), reducing capillary mean transit time and GCRs. mTBI is clinically diagnosed utilizing history/examination findings with no physiologic biomarkers. 3D TGSE (turbo-gradient spin-echo) pulsed arterial spin-labeling 3T MRI with 7 long inversion times (TIs) assessed the signal clearance of labeled protons 2800–4000 ms postlabeling in bifrontal, bitemporal, and biparietal regions within 7 days of mTBI and once clinically cleared to resume activities. The Sport Concussion Assessment Tool Version 5 (SKAT5) and Brief Oculomotor/Vestibular Assessment evaluated injured athletes’ cognitive function prior to MRIs. The pilot study demonstrated significant GCRs improvement (95% CI − 0.06 to − 0.03 acute phase; to CI—recovery CI 0.0772 to − 0.0497; *P* < 0.001 in frontal lobes; and parietal lobes (95% CI − 0.0584 to − 0.0251 acute; CI − 0.0727 to − 0.0392 recovery; *P* = 0.024) in 9 mTBI athletes (8 female, 1 male). Six age/activity-matched controls (4 females, 2 males) were also compared. mTBI disrupts the BBB, reducing GCR measured using the 3D ASL MRI technique. ASL MRI is a potential noninvasive biomarker of mTBI and subsequent recovery.

## Introduction

Traumatic brain injury (TBI) is a major worldwide health concern affecting an estimated 369 per 100,000 people^1–3^. Ninety percent of injuries are mild (mTBI), defined as a Glasgow Coma Scale score of 13–15/15. Sports-related injuries account for an estimated 17% of injuries; however, the number of cases is underestimated, as the epidemiologic data are derived from hospital admissions, excluding those managed conservatively^4^. All-cause and severity indirect and direct TBI costs were estimated to be $93 billion in 2019 in the US alone, not including lost wages and unemployment. The latter predominantly affects those with moderate or severe TBI^3^. The sequelae after even mild injury may include overt or subtle emotional, cognitive, and endocrinologic dysfunction^5,6^.

The diagnosis of sports-related acute mild TBI (mTBI) is reliant on cognitive and physical exam inventories such as the Sport Concussion Assessment Tool, 5th edition (SKAT5) obtained at the time of injury, and Vestibular Ocular Motor Screening (VOMS)^7,8^. If these tests are positive for a concussive injury, athletes are held out of related activity until symptoms abate and the above tests revert to baseline. Routine CT brain and anatomic MRI imaging studies are typically normal, ruling out structural injury in the vast majority of patients with mTBI, but are of little value in addressing the altered physiology due to damaged blood brain barrier (BBB)^9,10^. The measurable physiological effect on BBB integrity of mild TBI in humans can be studied directly looking for presence of leaked contrast agents or indirectly by assessing perfusion and flow dynamics. Dynamic contrast enhancement (DCE) employs gadolinium contrast directly assessing BBB leak^13,15^. Given the potential for contrast deposition within the nervous system with unknown long-term consequences, the technique is not suitable for multiple repeat studies. MRI diffusion tensor imaging (DTI) is sensitive to BBB fluid dynamics but is limited by long scan times of 1–2 h^10–12^. Functional MRI (fMRI) measuring regional changes in O_2_ utilization is an indirect measure of perfusion alterations post TBI but is limited by cost and long scan times, which make routine usage impractical^11–15^.

Our approach is to assess perfusion changes resulting from blood brain barrier (BBB) leakage inherent to acute TBI and determine if there is subsequent repair correlating with clinical recovery using noninvasive 3D ASL MRI^16^ BBB leakage is a direct result of blunt force injury and subsequent inflammatory changes that may develop^16–21^. The result is local alteration in the normal vascular perfusion affecting the capillary mean transit times (cMTT), which is the measure of time blood flows through the capillaries in a given brain region^22^. Glymphatic outflow is also directly reduced by BBB injury^16^. The net affect is delay in inflow of capillary blood with leakage of fluid which is then trapped.

**Figure 1:**
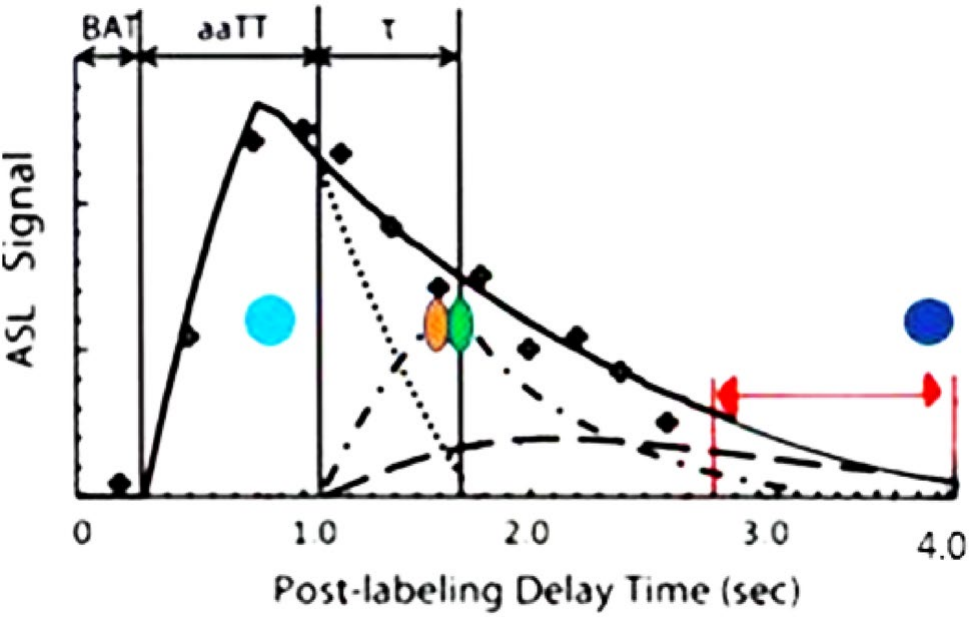
Components of Cerebral Perfusion versus Time. Composition of the ASL signal/time into its various components: arterial, capillary, and extra capillary spaces. TI (acquisition) times utilized in this study are between the red arrow limits. Note that the signal composition is capillary and extracapillary (interstitial) water; the latter is normally cleared by the glymphatic system. The 7 PLDs or times of acquisition in his study are between the red arrowheads.  total composite signal vs time  arterial signal contribution vs time  intracapillary signal contribution vs time  Extracapillary fluid signal contribution vs time. T1 times (63% signal decay) of major signal contributors indicated by the colored dots. Magenta dot = 800–850 ms T1 of white matter. Orange dot = 1650 ms T1 of blood. Green dot = 1700 ms T1 of gray matter (all values are for 3 T). Dk blue dot = 3800 ms T1 of water (CSF fluid); (all values are for 3 T). BAT = bolus arrival time; aaTT = artery-artery arrival time; τ = peak capillary arrival time. Adapted with publisher permission (Wiley) from reference 24.

The concept of glymphatic clearance rate (GCR) measures the time-dependent slope of the reduction in labeled proton signal in the late capillary phase of perfusion^23^ (Fig. 1). The constituent labeled proton signal in the late perfusion phase is from both labeled blood inflowing during the capillary mean transit time (cMTT) and the presence of residual labeled free fluid present in the interstitium by diffusion. Reduced GCRs (post-BBB injury) are caused by delayed cMTT and increased labeled free fluid trapped in the interstitium due to both BBB injury related leak and consequent reduced glymphatic outflow^10,16,17,22^. Simply put blood is slow to arrive, leaks in and can’t get out. Blood brain barrier leak results in delayed cMTT and disruption of glymphatic flow, both of which are demonstrable in the latter portion of the perfusion cycle by ASL MRI. Measuring the signal average of labeled protons within a region of interest (ROI) within the brain parenchyma can be graphed versus time. Using linear analysis, the slope of the signal strength over time is the GCRs which is normally negative (fluid flow out) but is flat or slightly positive in BBB injury (retained fluid)^10,16,17,22^. White matter fluid flow is delayed regionally by BBB leak to a greater extent than in associated gray matter^12,25^. 3D ASL MRI is a noninvasive and available sequence on most 3 T and above MRI scanners. By labeling protons in the neck, endogenous contrast is produced, and the signal can be measured at selected delay times (TI) post labeling (PLD) during the perfusion cycle^25–29^. By choosing long delay times at 2800–4000 ms, only the residual labeled protons from the delayed capillary phase of perfusion and free fluid are measured in the region of interest (ROI) (Fig. 1). The ROI, although hand drawn, encompasses a large region whose volume and shape are held constant, with the same slice chosen for each of the 7 determinations. Blood brain barrier leakage results in reduced GCRs compared to normal GCRs. The GCR reduction is not specific for head injury but occurs in any disease process damaging the BBB, such as Alzheimer’s disease^22,29–33^. By using this approach in a pilot study, we found reduced labeled proton clearance rates in subjects with Alzheimer’s disease (where BBB leak develops early in the process) compared to normal age-matched control subjects^27^. Given the presence of BBB leakage in mTBI, the possibility of extrapolating the ASL MRI technique was explored. The short scan time of under 20 min, low cost, and low safety risk (no gadolinium contrast) make this a potentially attractive diagnostic biomarker to investigate.

## Methods

### Study subject and control recruitment

In this case-controlled study, potential subjects between 18 and 30 years of age were recruited following acute mTBI. Nine varsity student athletes (1 male, 8 female, ages 19–22) with mTBI (direct cranial impact injuries only) and no other significant underlying disease or prior injury were studied within 1 week of injury and then again upon clinical recovery. The study commenced in late 2021 and concluded in the spring of 2023. All subjects gave written informed consent to participate in accordance with the Declaration of Helsinki. One subject had 2 head injuries within 3 weeks and was studied twice before the clinical recovery scan (but counted once in the data analysis). A control group of non-injured age- and activity-matched volunteers (2 males, 4 females, ages 19–23) was also obtained for comparison (Table 1). Four of the six non-injured volunteers were division one athletes, and two of the six students were former athletes and currently physically active.

**Table 1:** Data tabulation of injured and control groups.

Cohort (n) sex % distribution mean age (MA) (range)	ID #	Sport	Date of HI	SCAT5 symptom quantity	SCAT5 symptom severity	VOMS Δ*	Brain region affected	Days between injury and recovery scans
mTBI (9) M:F = 1:8 11.1%: 88.9% MA = 20.2 yrs (± 1.2)	1	S/DIVING	Nov-21	9	23	> 3**	BP, LF	56
	2	Cheer lead	Jan-22	10	22	> 3**	BF	27
	3	VB (HI X 2)	Jan-22	10	22	> 3**	BF, BP	28
			Feb-22	10	23	> 3**	BF, BP	
	4	WLAX	Feb-22	7	10	6	BF	28
	5	WLAX	Sep-22	14	42	> 3**	BT, BP	21
	6	MSOC	Sep-22	12	32	> 3**	BF	48
	7	WLAX	Feb-22	16	34	> 3**	BF	21
	8	FH	Oct-22	15	36	5	B(T,F,P)	17
	9	VB	Oct-22	10	20	6	LF	28
Control (6) M:F = 2:4 33.3%:66.7% MA = 21.8 yrs (± 2.1)	1	S/diving	None					
	2	SB	None					
	3	Basketball	None					
	4	VB	None					
	5	Runner	None					
	6	Track sprint	None					

*The higher post injury score compared with baseline indicates high likelihood of a cerebral concussion. **VOMS score ≥ 3 (a scale of 1–10 assesses headache, dizziness, nausea, and fogginess) supports a concussion diagnosis. Sensitivity = 64% specificity = 74%. The clinical symptom and evaluations were obtained at the time of injury or upon initial clinical evaluation prior to the acute injury MRI. All acute post injury scans were obtained within 7 days after injury. The clinical evaluations and progressive reinsertion in sport activity is tied to recurrence of symptoms. With recurrence of symptoms activity level is reduced and a longer period of convalescence is instituted ± additional testing, the latter more prevalent with more severe head injury. *S/diving* swim/diving, *VB* volleyball, *WLAX* women’s lacrosse, *MSOC* men’s soccer, *FH* field hockey, *SKAT 5* sport concussion assessment tool 5th edition, *VOMS* vestibular oculomotor screening. *B* bilateral *F* frontal, *P* Parietal, *T* temporal, *L* left *R* right.

Potential variables include location of injury, severity of impact, duration of altered consciousness or confusion, and evidence on FLAIR sequence of new or old structural injury. Potential subjects with chronic CNS disease, prior head injury or stroke, systemic inflammatory, heart or other chronic illnesses, nonremovable metal piercings or MRI incompatible implants, or claustrophobia were excluded from this study. Potential biases include exclusively studying a young athletic head injured subject pool with mild TBI. The former bias was justified by the otherwise healthy cohort reducing illness confounders, and the latter was justified by efforts to determine test sensitivity. The study size was extrapolated from our initial pilot study using ASL MRI, which demonstrated statistically significant differences (power of 0.8) in three Alzheimer disease subjects compared with age-matched controls^23^. Given the inciting presence of BBB leakage in both groups (AD and TBI), the subject numbers seemed justified for an exploratory pilot study.

Following an athletic injury that could precipitate an mTBI, an athletic trainer performed an initial evaluation using the SCAT 5. If these results were suggestive of a concussion, the athlete was further evaluated by a sports medicine physician within 48 h for a definitive diagnosis^5,34–36^. The physician reviewed the SCAT5, obtained a history, and performed a physical exam. The physical exam included a cranial nerve exam and, if warranted, a Vestibular Oculomotor Screening (VOMS) (Table 1). In addition, osteopathic screening and manipulative treatments were performed in some cases. Per convention, the injured athletes were removed from sport participation and instructed to employ as much cognitive rest as possible, including academic accommodations, limitations to screen-time, etc., along with strict physical rest. The athletic training staff followed their recovery progress and began a standard five-day return-to-play (RTP) protocol under physician supervision once the athlete was asymptomatic. After the athlete had successfully returned to their academic work and the RTP protocol was completed without symptom recurrence, they were medically cleared to resume full participation in their sport. No financial or other incentives were offered for participation. Table 1 Subject Demographics and Acute Injury SKAT5 and VOMS Scores.

### Imaging and data analysis methods

The contribution of each tissue component (white matter, gray matter, blood, and free fluid) within the region of interest at specific postlabeling delay times (PLD) of the total signal average is dependent on their respective T1 values (Fig. 1). Using their respective T1 values at 3T, and perfusion component data from Li et al. clearly show that the component signal in the late phase of perfusion is residual capillary flow plus residual labeled fluid in the region of interest^24^. The choice of timing of the 7 PLDs was based on this combined data, which minimized all but the 2 components desired: residual free water and labeled capillary fluid in the interstitium. Using the 3D TGSE PASL MRI protocol previously published, seven ASL sequences using TIs (time to inversion) starting at 2800 ms and at 200 ms intervals to 4000 ms were obtained^23^. In addition, an index FLAIR axial sequence was obtained. Scan Sequence details are as follows:

### 3D TGSE PASL MRI sequences

Our ASL sequences include: FOV 256X256, FOV phase 100%; TR 3830 ms; TE 16.40 ms; and TI 2800, 3000, 3200, 3400, 3600, 3800, 4000 ms. Additional parameters are TE 16.4 ms. (all sequences), TR 3830 with inversion time (TI) 2800; 3200; 3400; 3600; 3800; TR 4100 for inversion time TIs 4000. Acquisition time 2 min, 10 s per sequence except for the TI of 4000 with a scan time of 2 min, 19 s. Matrix 64X64X40; Averages 1 concatenations 1; Segments 16; Four averages (pairs); Bolus labeling 700 ms, FOV 256 mm × 256 mm. Post-inversion image acquisition time was 350 ms. Bolus labeling duration was 700 ms. Turbo factor was 12, and EPI factor was 21. Q2TIPS and vendor-supplied proprietary background suppression were used. A bandwidth of 2368/pixel and echo spacing of 0.57 ms were employed. Interleaved 40–4 mm image slices were obtained per sequence and voxel size was 2 × 2 × 5.5 mm3. Additional factors include distance factor 50%, Base resolution 64, Phase resolution 98%, and FoV phase 100%.

### Flair sequence

The flair sequence is TR 9000 ms; TE 84 ms; TI 2500; TI 2500 ms; Pixel volume 0.7 × 0.7 × 4 mm^3^.

Slice 4 mm interleaved, Fat Sat strong Scan time 3:20.

Total overall scan time was 16 min, 39 s.

**Figure 2:**
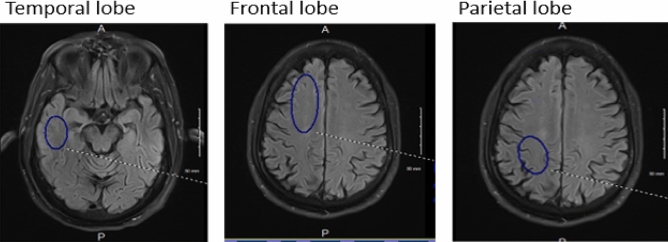
Selected brain regions chosen from the flair sequence reference scan for ROI signal averaging. The blue oval contains the region of signal average: temporal lobe 650 mm^2^; frontal lobe 1150 mm^2^; and parietal lobe 760 mm^2^. The volume, shape, and location were held constant with minimal intra and intersubject variation. Homologous contralateral determinations were also obtained. The FLAIR index scan and perfusion sequences were position locked such that the ROI was obtained from the same slice for all 7 perfusion images.

**Figure 3:**
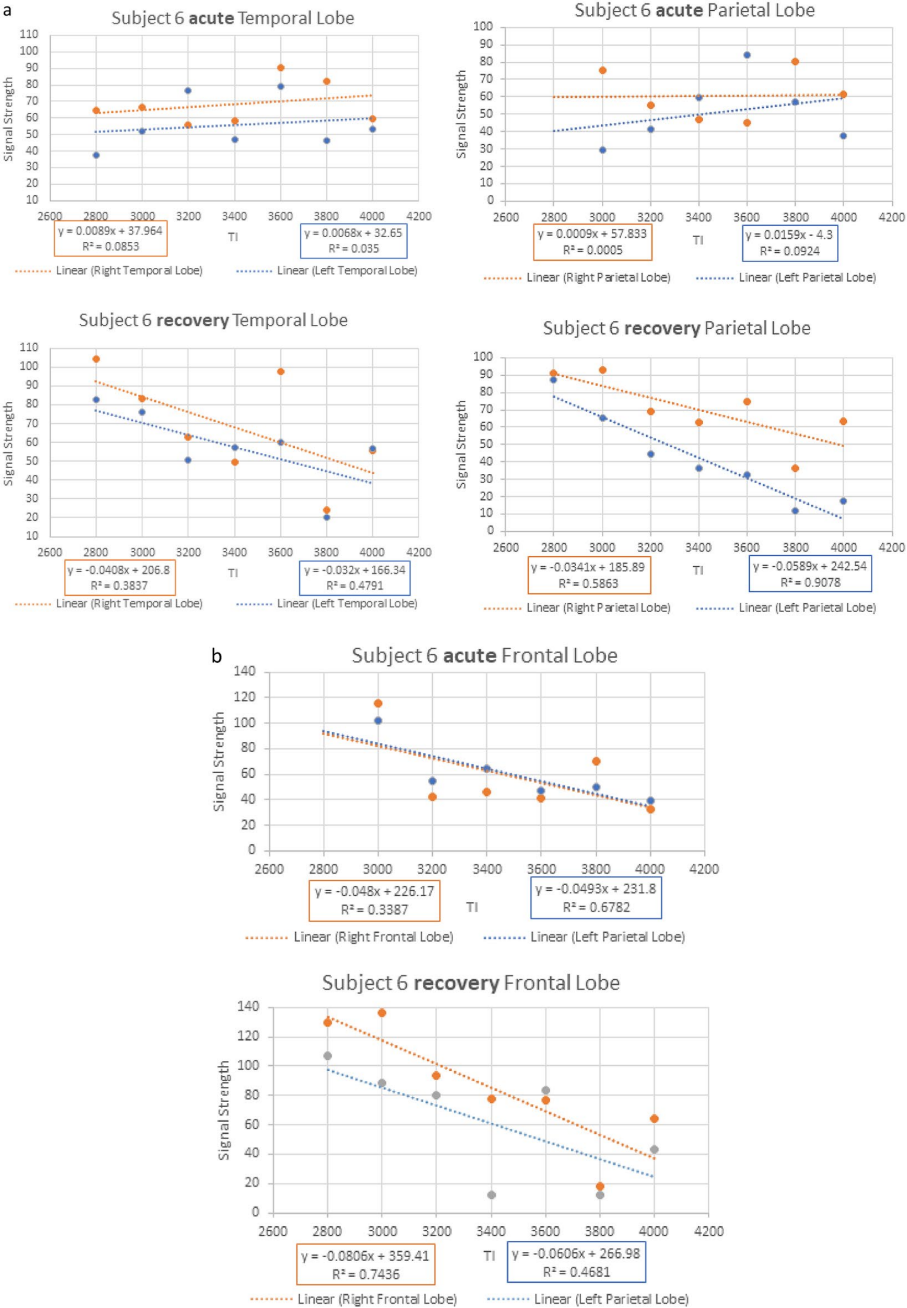
Example of signal averages vs. time for Subject 6. Example of signal averages vs. time. (**a**) Subject 6 Acute injury with reduced clearance rates (positive GCR slopes) affecting the bilateral Temporal and Parietal lobes in the upper two panels thus indicating delayed capillary inflow and diminished glymphatic outflow. Their respective recovery study results in the lower panel shows return to normal clearance (negative GCR slopes). Note the numeric GCR slope s for each location is in the color-coded boxes below the graph. (**b**) The unaffected Frontal lobes in subject 6 are noted showing post-acute injury above and recovery below with no change in the normal GCRs.

The total scan time was 18½ min. The 3D images were then formatted into 4 mm contiguous axial slices from which a single slice at the same level of all ASL sequences was chosen from the index flair axial sequence. Postprocessing was completed using position locked Perfusion and Flair images transferred to PACS and selected images were studied using the elliptical ROI tool (PACS). A slice-specific constant volume and location for each brain region were studied (4 mm axial slices). The bilateral homologous temporal lobes ROI was 650 mm^2^ individually obtained just above the temporal horn for all subjects. The bilateral homologous frontal lobes ROI was 1150 mm^2^ individually just above the lateral ventricles. The bilateral homologous parietal lobes ROI was 760 mm^2^ individually at the same level as the frontal lobe ROI but posterior to it (Fig. 2). By choosing a large ROI in each brain region and avoiding subarachnoid and ventricular spaces, we reduced potential sources of error related to contamination with extra parenchymal fluid and signal voxel-to-voxel signal variability. Signal averages were transferred to a spreadsheet and graphed vs time (Fig. 3). Since the data collection occurs in the late phase of the perfusion cycle (egress of blood and fluid), a close approximation by linear analysis can be applied with the slope of the line corresponding to the clearance of labeled protons^14,23^. The acute injury and recovery results were then pooled and compared statistically. All subjects were scanned in the midafternoon to early evening hours and had withheld alcohol and caffeine for the preceding three hours before their study.

### Statistical methods

Gender and age were summarized in each group using frequency distributions and summary statistics. ROIs were analyzed in separate models. Because each subject had nested bilateral measures of GCR slope that were repeated under injury and recovery conditions, to compare the GCR slopes for the two conditions within mTBI subjects, a linear mixed effects model with random intercepts for the subject and lateral sides was used initially. After finding minimal variability between the two lateral measures within each subject for all three ROIs, this random intercept was removed from the model (leaving only a random intercept for subjects). Similarly, when comparing mTBI subjects to control subjects, a linear mixed effects model was used with an intercept for each subject to account for bilateral nesting of GCR slopes within subjects. Visualization of model residuals indicated approximate normality in all cases. IBM SPSS v 29 was used for all statistical analyses.

## Results

See appendix for the full clearance rate results for all subjects (acute injury and recovery as well as controls).

The results from the three linear mixed effects models comparing the *acute* and *recovery* slopes of the line correspond to the clearance of labeled protons over time (Table 2). This table includes estimated marginal means, differences, standard errors, and statistical test results.

**Table 2:** Comparison of Acute injury GCR with Recovery GCR.

Lobe	Time	Mean	SE(M)	Difference	95% CI (Diff)		t	df	*p*
Temporal	Acute	− 0.0545	0.0082	0.0052	− 0.0115	0.0220	0.64	28.8	0.526
	Recovery	− 0.0598	0.0084						
Frontal	Acute	− 0.0462	0.0062	0.0172	0.0083	0.0261	3.96	28.1	< 0.001
	Recovery	− 0.0635	0.0062						
Parietal	Acute	− 0.0417	0.0075	0.0142	0.0021	0.0264	2.39	28.1	0.024
	Recovery	− 0.0559	0.0076						

Table 2 shows that there are statistically significant differences at the 0.05 level of significance with associated confidence intervals for both frontal and parietal lobes (pooled results); in both cases, the acute slopes are positive or less negative than the recovery slopes. The pooled temporal lobe results for acute and recovery showed no evidence that the measures were different indicating a less frequent site of injury within the cohort of injured players (except subject 6).

**Figure 4:**
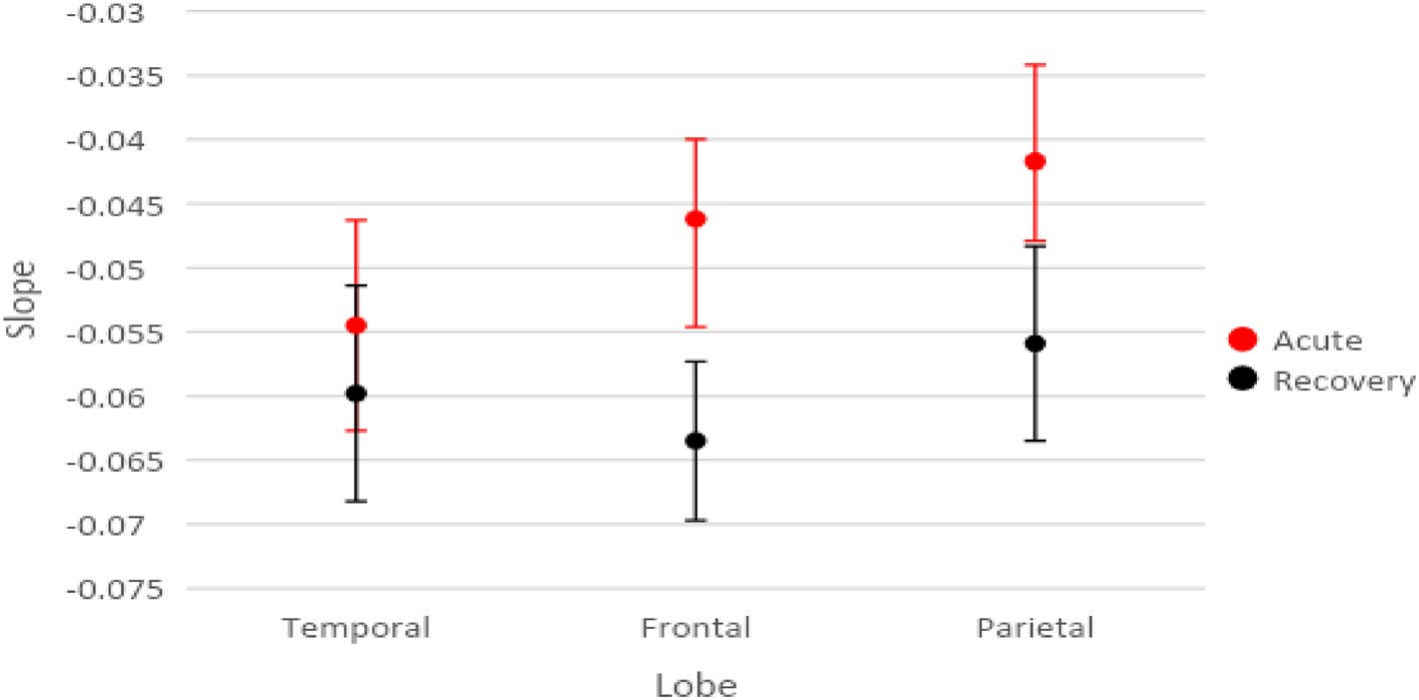
Modeled Means and Standard Error Bars by Time and Lobe for the acute injury and recovery scans comparing GCRs.

Figure 4 includes the means and standard error bars of the slopes for each of the three lobes combining homologous GCRs for both acute and recovery scans in the injured group.

Table 3 shows the results from the three linear mixed effects models comparing the slopes of the acute phase of the mTBI group to the control group.

**Table 3:** Comparison of acute injury GCR.

Lobe	Time	Mean	SE(M)	Difference	95% CI (Diff)		t	df	*p*
Temporal	mTBI	− 0.0525	0.0098	0.0109	− 0.0224	0.0442	0.70	14.1	0.494
	Control	− 0.0634	0.0121						
Frontal	mTBI	− 0.0455	0.0073	0.0215	− 0.0036	0.0465	1.85	13.0	0.087
	Control	− 0.0670	0.0090						
Parietal	mTBI	− 0.0416	0.0082	0.0230	− 0.0050	0.0520	1.80	12.9	0.095
	Control	− 0.0651	0.0101						

From Table 3, with controls showed there are no results that are statistically significant at the 0.05 level of significance (frontal *p* = 0.087, parietal *p* = 0.095 temporal = 0.494). Although the affected parietal and frontal lobe clearance rates did not overlap with those of normal controls, the differences did not reach statistical significance. The infrequently affected temporal lobe in the pooled data showed no evidence that the measures were different from the control group clearance rates.

**Figure 5:**
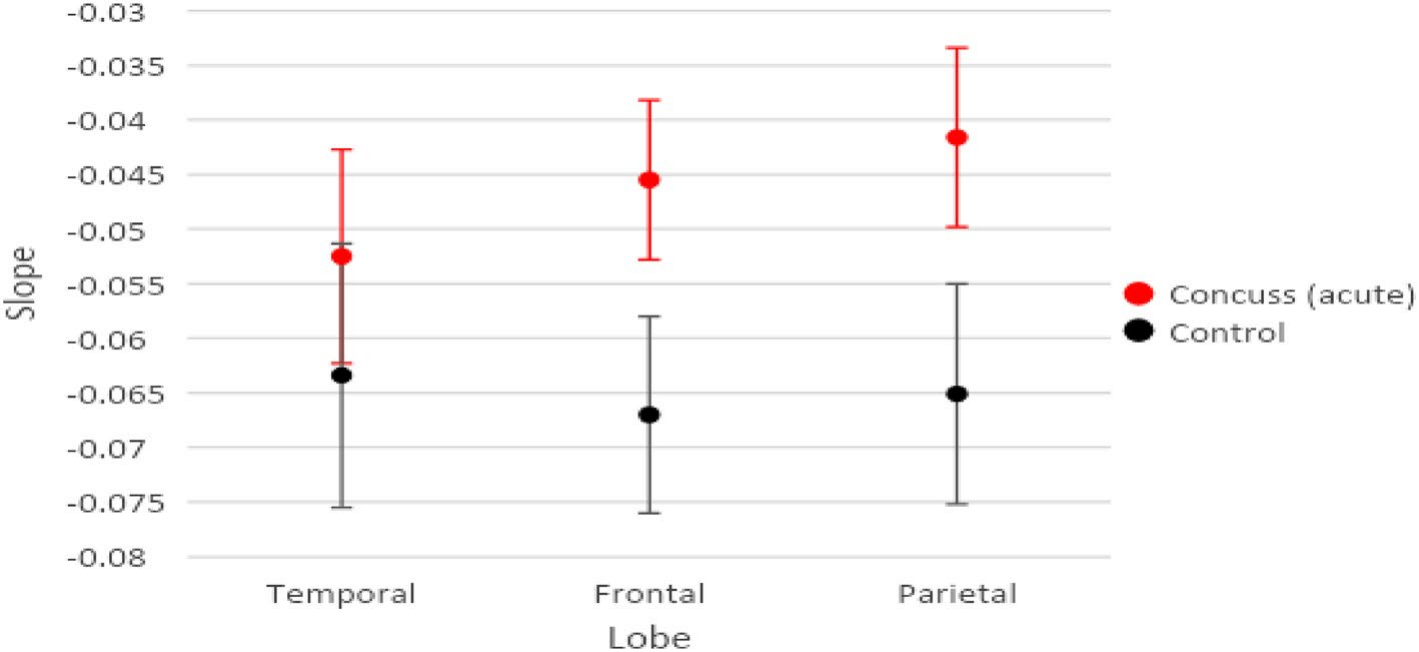
Modeled Means and Standard Error Bars by Group and Lobe comparing scans post-acute injury GCRs with controls.

Figure 5 includes the means and standard error bars of the slopes for the acute scans of injured subjects compared to the control group in each of the three brain regions combining homologous GCRs s.

Table 4 shows the results from the three linear mixed effects models comparing the slopes of the recovered phase of the mTBI group to the control group.

**Table 4:** Comparison of recovery GCR with control group.

Lobe	Time	Mean	SE(M)	Difference	95% CI (Diff)		t	df	*p*
Temporal	mTBI	− 0.0598	0.0058	0.0036	− 0.0161	0.0233	0.40	13.0	0.696
	Control	− 0.0634	0.0071						
Frontal	mTBI	− 0.0635	0.0082	0.0035	− 0.0245	0.0315	0.27	13.0	0.791
	Control	− 0.0670	0.0101						
Parietal	mTBI	− 0.0559	0.0054	0.0091	− 0.0093	0.0276	1.07	13.0	0.304
	Control	− 0.0651	0.0066						

Table 4 there are no results that are statistically significant at the *p* ≤ 0.05 level of significance. Thus, **recovery** clearance rates in the acute injury affected parietal, frontal, and temporal lobes showed no evidence that the measures are different from the control group.

**Figure 6:**
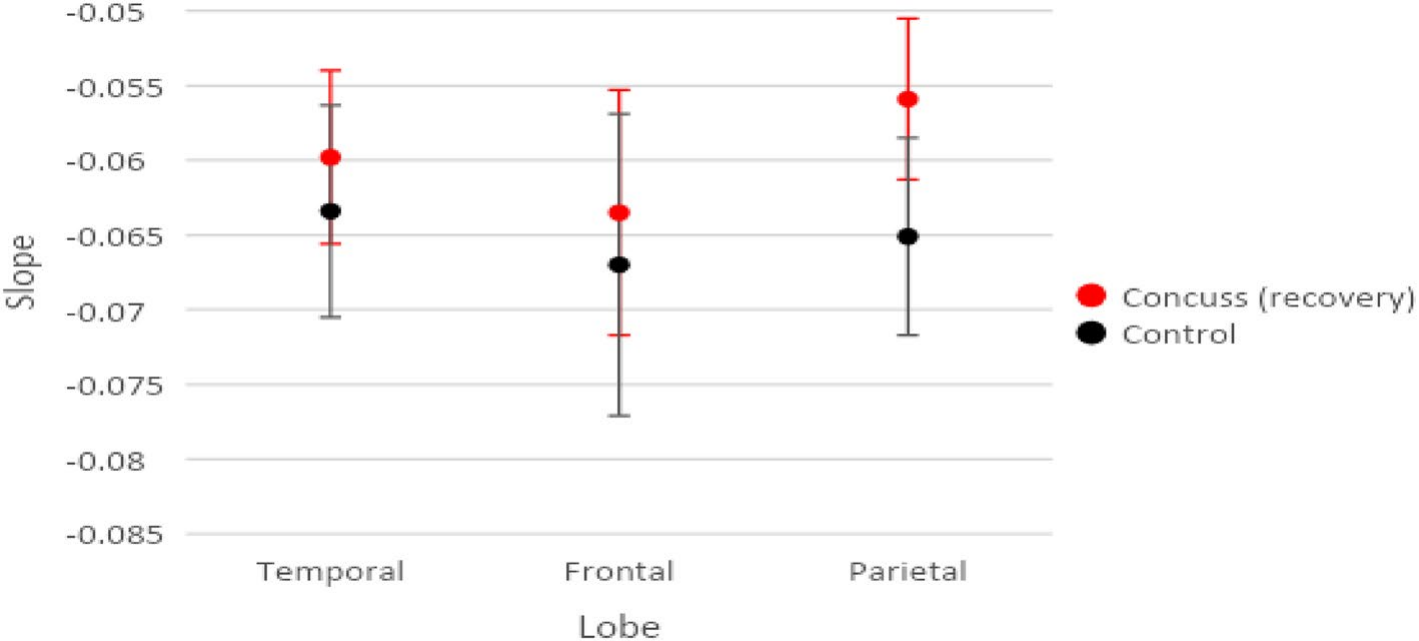
Modeled Means and Standard Error Bars by Group and Lobe comparing post recovery scans and control group GCRs.

Figure 6 includes the means and standard error bars of the recovery scan slopes for injured subject compared with control subjects for each of the three brain regions combining homologous GCRs.

## Discussion

This pilot study using ASL MRI within one week of acute mild head injury in student athletes with Glasgow Coma Scale scores of 13–15 showed a localized reduction in clearance rates (GCRs) of labeled protons^37^. When clinically cleared to return to their sport, statistically significant improvements in clearance rates were noted, suggesting good sensitivity of the technique. The GCR improvement likely corresponds to repair of the BBB leak inherent to ACI. The advantages of this technique include low cost, no exogenous contrast agents, MRI sequence availability, and short scan times. A larger study is warranted to further validate this novel approach for widespread use as a biomarker in ACI and recovery. With newer multilabeling ASL techniques available, the scan time may be significantly reduced to only 5–6 minutes^38^. The advantage of this method is the use of intrinsically labeled protons in blood altered only by spin orientation, which does not disturb the underlying physiology. Gadolinium contrast techniques such as DCE require diffusion calculations and are invasive. Furthermore, ASL MRI can be repeated multiple times given the absence of extrinsic contrast, which in contact sports activities may provide guidance otherwise missing as to when and if an athlete can return safely to sport^35,36^. Additionally, the technique may provide an outcome measure for various future therapeutic endeavors to repair BBB leakage in more severe head injury and a variety of diseases where it is impaired once the repair pathways are more fully delineated.

Of interest are the brain injury locations in this study, showing that the most common acute injuries were the frontal and parietal lobes sparing the temporal lobes, with the exception of subject 5 where it was involved. The mild degree and site of impact likely explains this in the majority of the studied subjects. Subject 3 is of interest as she was injured twice within 6 weeks and two acute injury scans showed consistent pattern of reduced GCRs which ultimately recovered. Although her data from both injuries were included only once in the data analysis, these results demonstrated consistency of this technique in acute post injury evaluation.

Limitations of our study include a small sample size (pilot study) and underrepresentation of male athlete volunteers. The latter is in part related to a higher risk of concussion in young women athletes related to several factors, such as sex hormones and physiologic changes^2,26,34^. In addition, male athletes were less likely to volunteer. The small number of normal controls may explain the lack of a significant difference in the clearance rates compared with the acute injury results. Additionally, the mild nature of head injuries may directly correlate with a less severe reduction in clearance rates. A larger study including more serious head injury patients would be a logical next step. In the absence of clearance rate recovery in more severe HI, anticipated cognitive neurologic deficits could be predicted, and appropriate rehabilitation measures could be applied. Technical issues regarding low signal strength are compensated by averaging a large field of view in each of the brain regions studied and obtaining multiple time delay data points in identical anatomic locations.

This pilot study shows the potential of adopting 3D ASL MRI with further validation in the evaluation of ACI and presumed recovery as an available, noninvasive, inexpensive, and efficient reproducible objective biomarker. It’s use could easily be extrapolated to other brain diseases where the blood brain barrier dysfunction is integral in both identifying early disease and assessing outcomes of future restorative treatments.

### Supplementary Information

Supplementary Information.

## Data Availability

All data generated or analyzed during this study are included in this published article [and its supplementary information files].

## References

[1] Maas AIR (2022). Traumatic brain injury: Progress and challenges in prevention, clinical care, and research.. Lancet Neurol..

[2] Forrest RHJ (2018). Mild traumatic brain injury in New Zealand: Factors influencing post-concussion symptom recovery time in a specialised concussion service. J. Prim. Health Care.

[3] Frieden, T. R., Houry, D. & Baldwin, G. Traumatic brain injury in the United States: epidemiology and rehabilitation. CDC NIH Rep to Congr 1–74 (2015).

[4] Williams RM (2015). Concussion recovery time among high school and collegiate athletes: A systematic review and meta-analysis. Sports Med..

[5] Polinder S (2018). A multidimensional approach to post-concussion symptoms in mild traumatic brain injury. Front. Neurol..

[6] Lo J, Chan L, Flynn S (2021). A systematic review of the incidence, prevalence, costs, and activity and work limitations of amputation, osteoarthritis, rheumatoid arthritis, back pain, multiple sclerosis, spinal cord injury, stroke, and traumatic brain injury in the United States: A 2019 update. Arch. Phys. Med. Rehabil..

[7] Dessy, A. M., *et al.* Review of assessment scales for diagnosing and monitoring sports-related concussion. *Cureus* **9**(12) (2017). 10.7759/cureus.1922 PMC5802754 29456902

[8] Gallet B (1989). Evaluation of pulmonary arterial hypertension by Doppler echocardiography in chronic respiratory insufficiency. Arch. Mal. Coeur Vaiss..

[9] Wintermark M (2015). Imaging evidence and recommendations for traumatic brain injury: conventional neuroimaging techniques. J. Am. Coll. Radiol..

[10] Elschot EP (2021). A comprehensive view on MRI techniques for imaging blood-brain barrier integrity. Investig. Radiol..

[11] Mavroudis I (2022). Post-concussion syndrome and chronic traumatic encephalopathy: Narrative review on the neuropathology, neuroimaging and fluid biomarkers. Diagnostics.

[12] Kim E (2022). A systematic review and data synthesis of longitudinal changes in white matter integrity after mild traumatic brain injury assessed by diffusion tensor imaging in adults. Eur. J. Radiol..

[13] Eierud C (2014). Neuroimaging after mild traumatic brain injury: Review and meta-analysis. NeuroImage Clin..

[14] Pollock JM (2009). Arterial spin-labeled MR perfusion imaging: Clinical applications. Magn. Reson. Imaging Clin. N. Am..

[15] Gordon Y (2014). Dynamic contrast-enhanced magnetic resonance imaging: Fundamentals and application to the evaluation of the peripheral perfusion. Cardiovasc. Diagn. Ther..

[16] Churchill NW (2017). The first week after concussion: Blood flow, brain function and white matter microstructure. Neuroimage Clin..

[17] Abbott NJ (2018). The role of brain barriers in fluid movement in the CNS: Is there a ‘glymphatic’system?. Acta Neuropathol..

[18] Profaci, C. P. *et al*. The blood–brain barrier in health and disease: Important unanswered questions. *J. Exp. Med.* **217**(4) (2020). 10.1084/jem.20190062 PMC7144528 32211826

[19] Sulhan S (2020). Neuroinflammation and blood–brain barrier disruption following traumatic brain injury: Pathophysiology and potential therapeutic targets. J. Neurosci. Res..

[20] Hu Y, Tao W (2021). Microenvironmental variations after blood–brain barrier breakdown in traumatic brain injury. Front. Mol. Neurosci..

[21] Chow BW, Chenghua G (2015). The molecular constituents of the blood–brain barrier. Trends Neurosci..

[22] Dave RS, Jain P, Byrareddy SN (2018). Functional meningeal lymphatics and cerebrospinal fluid outflow. J. Neuroimmune Pharmacol..

[23] Joseph CR (2020). Pilot study utilizing MRI 3D TGSE PASL (arterial spin labeling) differentiating clearance rates of labeled protons in the CNS of patients with early Alzheimer disease from normal subjects. Magn. Reson. Mater. Phys. Biol. Med..

[24] Li K-l (2005). Four-phase single-capillary stepwise model for kinetics in arterial spin labeling MRI. Magn. Reson. Med. Off. J. Int. Soc. Magn. Reson. Med..

[25] Yu L (2022). Perivascular spaces, glymphatic system and MR. Front. Neurol..

[26] Crasta JE (2022). Altered white matter diffusivity and subtle motor function in a pilot cohort of adolescents with sports-related concussion. Brain Injury.

[27] Grade M (2015). A neuroradiologist’s guide to arterial spin labeling MRI in clinical practice. Neuroradiology.

[28] Detre JA (1992). Perfusion imaging. Magn. Reson. Med..

[29] Joseph CR (2021). Utilizing 3D arterial spin labeling to identify cerebrovascular leak and glymphatic obstruction in neurodegenerative disease. Diagnostics.

[30] Iturria-Medina Y (2016). Early role of vascular dysregulation on late-onset Alzheimer’s disease based on multifactorial data-driven analysis. Nat. Commun..

[31] Madsen LS (2023). Capillary dysfunction correlates with cortical amyloid load in early Alzheimer’s disease. Neurobiol. Aging.

[32] Chagnot A, Barnes SR, Montagne A (2021). Magnetic resonance imaging of blood–brain barrier permeability in dementia. Neuroscience.

[33] Joseph CR (2020). Novel MRI techniques identifying vascular leak and paravascular flow reduction in early Alzheimer disease. Biomedicines.

[34] Davis-Hayes C (2017). Sex-specific outcomes and predictors of concussion recovery. JAAOS-J. Am. Acad. Orthop. Surg..

[35] Chen J-K (2007). A validation of the post-concussion symptom scale in the assessment of complex concussion using cognitive testing and functional MRI. J. Neurol. Neurosurg. Psychiatry.

[36] Arrieux JP, Wesley RC, Angelica PA (2017). A review of the validity of computerized neurocognitive assessment tools in mild traumatic brain injury assessment. Concussion.

[37] Nelson, L. D.,* et al.* Recovery after mild traumatic brain injury in patients presenting to US level I trauma centers: a transforming research and clinical knowledge in traumatic brain injury (TRACK-TBI) study. *JAMA neurol.* **76**(9), 1049–1059 (2019). 10.1001/jamaneurol.2019.1313 PMC6547159 31157856

[38] Yu, F., *et al.* Research applications of cerebral perfusion magnetic resonance imaging (MRI) in neuroscience. *PET/MR: Funct. Mole. Imag. Neurolog. Dis. Neurosci.* 79–92 (2023).

